# Comparing length of stay between adult patients admitted to the intensive care unit with alcohol withdrawal syndrome treated with phenobarbital versus lorazepam

**DOI:** 10.62838/jccm-2026-0020

**Published:** 2026-07-27

**Authors:** Mary C Hoye, Sorin Danciu, Marc A Jacobs, Brad J Glavan

**Affiliations:** Providence Portland Medical Center, Portland, OR, USA; The Oregon Clinic, Portland, OR, USA

**Keywords:** quality improvement, substance withdrawal syndrome, lorazepam, phenobarbital, alcoholism

## Abstract

**Objective:**

Review the use of phenobarbital and lorazepam to treat alcohol withdrawal syndrome (AWS) in the ICU, compare length of stay, examine medication use trends, implement provider training, and evaluate outcomes post-training.

**Methods:**

Design - Retrospective observational study, Quality improvement. Setting - Tertiary care hospital with 36 ICU beds. Patients - Adults admitted to the ICU and placed on clinical institute withdrawal assessment (CIWA) protocol. Patients with epilepsy were excluded.

**Results:**

During the 34-month baseline period, 713 patients were admitted to the ICU with alcohol use disorder (AUD) on CIWA, without epilepsy. 189 patients were treated with phenobarbital, 460 patients received only lorazepam, 64 patients received neither medication. All but 2 of the patients who received phenobarbital also received lorazepam. Compared to phenobarbital, lorazepam-only patients had shorter ICU LOS (p<0.001, 95% CI −2.36, −1.32) but higher mortality 13.91% vs. 4.76% (p=0.0008). We then developed and provided a training (which we refer to as an “intervention”) to all ICU providers encouraging consistent use of phenobarbital in the ICU when appropriate. During the 3-month post-intervention period 44 patients were admitted to the ICU with AUD on CIWA protocol. Of the 44 patients: 26 received phenobarbital, 12 received only lorazepam, 6 received neither medication. Significantly more patients were treated with only phenobarbital (57.69%)) compared to baseline (1.06%). Compared to patients treated with phenobarbital, patients treated with only lorazepam had a significantly higher mortality rate (33.33% vs. 7.69%, p=0.04).

**Conclusions:**

We found significant variability in the use of phenobarbital and lorazepam for treatment of AWS in the ICU. After a quality improvement training for ICU physicians, there was less frequent concurrent use of benzodiazepines and barbiturates, no difference in ICU or hospital LOS, and significantly lower mortality rate for those treated with phenobarbital.

## Introduction

Alcohol withdrawal syndrome (AWS) is a serious, sometimes fatal consequence of abrupt discontinuation of alcohol use in people with alcohol use disorder (AUD). In the United States, AUD is very common in patients admitted to hospitals, some studies estimate nearly 40% of people admitted to hospitals have AUD [1]. It is estimated that 7–8% of patients admitted with AUD will experience AWS, and as a result will experience longer hospitalizations, especially if the withdrawal is severe [1, 2]. Therefore, the safe and effective treatment of AWS is of interest to improve patient safety and decrease hospital length of stay (LOS). First-line treatment of AWS is with benzodiazepines (as recommended by the only current professional guidelines from the American Society of Addiction Medicine (ASAM)) which are agonists at gamma-amino butyric acid (GABA) receptors in the central nervous system [3,4,5]. However, due to the high incidence of AWS in hospitalized patients and therefore the high burden of care on hospital systems, there is significant interest in identifying more effective treatments for AWS. Phenobarbital (PB) is one such alternative treatment option, which, like benzodiazepines, agonizes GABA receptors but also suppresses glutamate receptors resulting in less of the activating symptoms of withdrawal such as delirium and agitation. In addition, phenobarbital has a much longer half-life than benzodiazepines which can decrease the need for additional medication dosing and has a linear pharmacokinetic profile, which makes its metabolism more predictable than benzodiazepines. ASAM guidelines state that phenobarbital may be used for severe withdrawal either alone or as adjunct medication with benzodiazepines by professionals familiar with PB use. There are few contraindications to PB, though like benzodiazepines caution should be used in patients with marked hepatic impairment [6]. While patients with AUD may have comorbid mental health and medical conditions, ASAM guidelines do not recommend different medication management of AWS for different populations (e.g patients with psychiatric illness do not need to be managed differently) [3].

Several studies have demonstrated the safety of phenobarbital in treatment of alcohol withdrawal [7,8,9]. Although a limited number of trials compare phenobarbital alone to lorazepam, one retrospective cohort study showed fewer ICU admissions and shorter LOS using fixed dose phenobarbital plus lorazepam compared to lorazepam alone [10]. Due to its demonstrated safety and efficacy in AWS, phenobarbital is being used more frequently both in emergency departments and inpatient settings as a safe and potentially more effective treatment for patients with AWS but there remains no consensus regarding when and how to use phenobarbital for AWS. ASAM guidelines note the need for large comparison studies of phenobarbital versus lorazepam for AWS management.

At our community based academic hospital, protocol-based treatment of patients with AWS admitted to the ICU allows the option to use lorazepam or phenobarbital. Treatment of AWS in the ICU tends to be provider-specific, using different combinations and dosages of phenobarbital, benzodiazepines, and other sedatives. The variability in clinician approach to AWS treatment presented an opportunity to review admissions to the ICU to determine if there was a difference in LOS between patients admitted to the ICU who received benzodiazepines versus those who received phenobarbital for AWS and to compare complication rates between the two groups.

Following review of the data from this baseline period, we developed an initiative to standardize care by providing a training (which will be called an “intervention”) to all ICU physicians promoting the use of an existing order set for the safe and effective use of phenobarbital in the ICU with the goal of maintaining patient safety while decreasing length of stay and complications.

## Materials and methods

To procure data, we leveraged Structured Query Language (SQL) to interact with the Providence Clarity Database, also known as the EPIC database. In addition, we used visualization software, Power BI, to enhance the efficiency of data filtering. We queried the database during the baseline period from 1/1/2019 through 10/17/2022, and again after physician training from 7/1/23 through 10/1/23.

In our primary query, we focused on gathering patient encounter details. This query contained several Common Table Expressions (CTEs). These CTEs included encounter details, ICU stays, Ventilator encounter, Phenobarbital Medical Administration Record (MAR), Phenobarbital given total, Lorazepam MAR, Lorazepam given total, Dexmedetomidine MAR, and Dexmedetomidine given total. Each CTE contained the same unique primary key, PAT_ENC_CSN_ID, which we used to merge the data tables via left joins. Secondary queries were used to help filtering via the visualization software. These included encounter diagnosis, encounter alcohol withdrawal related diagnosis, cirrhosis diagnosis, and CIWA scores associated with each encounter. Patients with a diagnosis of epilepsy were excluded.

Data were filtered to select for different groups of patients (ICU patients who received phenobarbital, ICU patients who received lorazepam, ICU patients who were mechanically ventilated). Data for the different groups were downloaded as Excel files. Excel was used to calculate mean hospital and ICU LOS. Mann-Whitney U tests were used to determine differences between mean LOS, and paired t-tests were used to determine all other differences between means. No adjustments were made for multiple comparisons.

The intervention involved meeting initially with several ICU physicians to develop a training to promote the use of a phenobarbital AWS order set available within the electronic medical record. We then met virtually with all the ICU attending physicians to provide the training promoting the use of the phenobarbital order set in the ICU for all ICU patients admitted with AWS unless phenobarbital was contraindicated based on patient factors (e.g. advanced liver disease). During this meeting, there was time for discussion of any concerns about phenobarbital use. After agreement to use the phenobarbital order set from ICU attendings, we provided training to each ICU resident physician and intern physician before they were scheduled to work in the ICU during the post-intervention period to promote the use of the phenobarbital order set. Training used screen shots detailing how to access and correctly use the order set and requested providers preferentially use the order set if a patient was being admitted to the ICU for treatment of alcohol withdrawal. After the training, we reviewed the use of phenobarbital and lorazepam in the ICU for a period of 3 months from 7/1/2023–10/1/2023, which was sufficient time for all providers to work in the ICU.

Our study proposal was reviewed by the Providence Hospital’s Institutional Review Board and was determined to be not human research.

## Results

Over 4 years, increasing amounts of phenobarbital were used, the average amount of lorazepam decreased, average hospital LOS decreased, and there was no significant change in ICU LOS.

While this study took place over 4 years (cumulative data in Table 1), the baseline period was limited to 34 months to limit confounding from new hospital policies regarding treatment of AWS which were implemented during this time. The post-intervention period was set to 3 months which allowed time for all providers to work in the ICU.

**Table 1.** Trends in average doses of phenobarbital and lorazepam, hospital LOS and ICU LOS during the 4 complete years in which the study was completed.

	**Total ICU patients**	**Average PB^1^ (mg/kg)**	**Average Lor^2^ (mg)**	**Hospital LOS^3^ (days)**	**ICU LOS (days)**
2019	220	1.65	72.46	12.01	4.98
2020	215	3.34	72.38	15.47	5.017
2021	148	2.10	48.4	14.91	6.26
2022	153	4.5	45.45	12.6	6.32

1. PB (phenobarbital);

2. Lor (lorazepam);

3. LOS (length of stay)

Patients included in the study were predominantly male (Table 2). Maximum CIWA score was significantly higher in those who were treated with phenobarbital in both the baseline and post-intervention periods.

**Table 2.** Demographics of patients included in the study during the baseline period and post-intervention period. P values reported from paired t-tests.

	**Baseline**		**p**	**Post-intervention**		**p**
	
	**PB**	**Lor**		**PB**	**Lor**	
N	189	460		26	12	
Age	51	58.6	<0.001	49.54	57	0.12
Female	35 (18.5%)	139 (30.2%)		6 (23.1%)	1 (8.3%)	
Male	154 (81.5%)	321 (69.8%)		20 (77.0%)	11 (91.7%)	
Max CIWA^1^	58	48	<0.001	53	22	0.01
Avg^2^ CIWA	32	18		23	15	
Cirrhosis (n)	7	18		0	0	
Avg PB^3^ (mg/kg)	11.44	na		15.6	na	0.10
Avg Lor^4^ (mg)	142	30		30.9	11	0.22
Dex^5^ (n received)	112 received (59.26%)	113 received (24.57%)		na	na	
Avg Dex (mcg/kg)	34.21	14.92		na	na	

CIWA (Clinical institute of withdrawal assessment);

Avg (average);

PB (phenobarbital);

LOR (lorazepam);

Dex (dexmedetomidine)

### Baseline

**Fig. 1.**
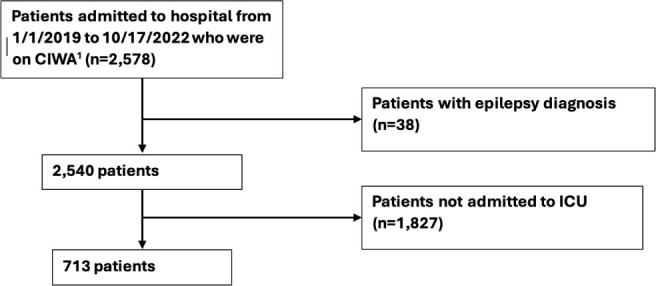
Patient inclusion criteria for baseline period (1/1/2019 through 10/17/2022). ^1^ CIWA (clinical institute of withdrawal assessment)

Thirty-four months of admission data was reviewed at one 483 bed tertiary care hospital with 36 intensive care beds. A total of 2,578 patients were admitted to the hospital between January 1, 2019 and October 17, 2022 who were placed on CIWA precautions. 38 patients were excluded from analysis due to epilepsy or other seizure disorder diagnosis. Of the 2,540 admitted patients placed on CIWA without epilepsy 713 were admitted to the Intensive Care Unit (ICU) and they had a mean hospital LOS of 13.80 days (median 8.24, SD 18.44), and a mean ICU LOS of 5.51 days (median 3.12, SD 7.25) (Figure 1).

Of the 713 ICU patients:

– 189 patients (26.51%) received phenobarbital and had a mean hospital LOS of 12.83 days (median 8.99 days, SD 12.44) and a mean ICU LOS of 7.33 days (median 4.91, SD 8.35), with an average dose of phenobarbital of 11.44 mg/kg (actual body weight), average total phenobarbital amount: 899.81mg. All but 2 of the patients who received phenobarbital also received lorazepam (average amount of 142.81 mg), (Table 3). 7 of the patients who received phenobarbital had a diagnosis of cirrhosis.
– 460 patients (64.52%) received only lorazepam and had a mean hospital LOS of 14.74 days (median 8.04 days, standard deviation 21.01), and a mean ICU LOS of 5.09 days (median 2.78, SD 7.05), with an average amount of lorazepam of 30.12mg, (Table 3). 64 patients received neither phenobarbital nor lorazepam. 18 of the patients who received lorazepam had a diagnosis of cirrhosis.

**Table 3.** Patients treated with phenobarbital and lorazepam compared with those treated with only lorazepam in the ICU during the baseline (top two rows) and post-intervention (bottom two rows) periods. LOS p-values were obtained from Mann-Whitney U tests; all other p-values are from paired t tests.

	**n**	**Hospital LOS^3^ (mean days +/−SD)**	**P-value**	**ICU LOS (mean days +/−SD)**	**P-value**	**Medication Amount**	**Mechanical Vent**	**P-Value**	**Discharge Alive (n)**	**P-Value**
PB^1^	189 (26.51%)	12.83 (+/− 12.44)	0.05 (95%CI: −2.21, 0.0014)	7.33 (+/− 8.35)	p<0.001 (95% CI: −2.36, −1.32)	11.44mg/kg	59 (31.22%)	0.65	180 (95.24%)	0.0008
Lor^2^	460 (64.52%)	14.74 (+/− 21.01)		5.09 (+/− 7.05)		30.12mg	152 (33.04%)		396 (86.09%)	

PB (post intervention)	26 (59.01%)	6.14 (+/− 8.58)	0.12 (95% CI: −1.21, 15.29)	3.77 (+/− 3.59)	0.13 (95%CI: −0.29, 4.28)	15.60mg/kg	8 (18.18%)	0.94	24 (92.31%)	0.04
Lor (post intervention)	12 (27.27%)	16.26 (+/− 12.67)		9.89 (+/− 13.00)		11.17mg	4 (9.09%)		8 (66.67%)	

PB (phenobarbital);

Lor (lorazepam);

LOS (length of stay)

**Fig. 2.**
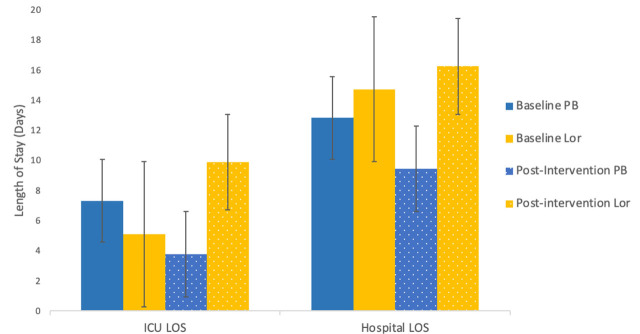
Mean ICU and hospital lengths of stay in patients treated with phenobarbital (PB) or lorazepam (Lor) +/− 1SD during the baseline and intervention periods. ICU LOS were significantly longer for those received PB compared to those who received lorazepam at baseline, but had no significant difference after the intervention. Hospital LOS was not significantly different during the baseline or intervention periods. (Mann-Whitney U testing used to test differences between mean LOS).

ICU patients who received phenobarbital had a significantly longer ICU LOS than those who received only lorazepam (p<0.001, 95% CI: −2.36, −1.32), but no significant difference in hospital LOS (p=0.05, 95% CI −2.21, 0.0014), Figure 2. When the patients with a diagnosis of cirrhosis in both the phenobarbital and lorazepam groups were excluded from the analysis, the ICU LOS remained significantly longer for the phenobarbital group (p<0.001, 95%CI: 1.29, 2.37) and there was no significant difference in hospital LOS (p=0.08, 95% CI: −0.13, 2.14).

The mean maximum CIWA score for those treated with phenobarbital was significantly higher (32) than for those treated with lorazepam (18, p=0.011).

**Fig. 3.**
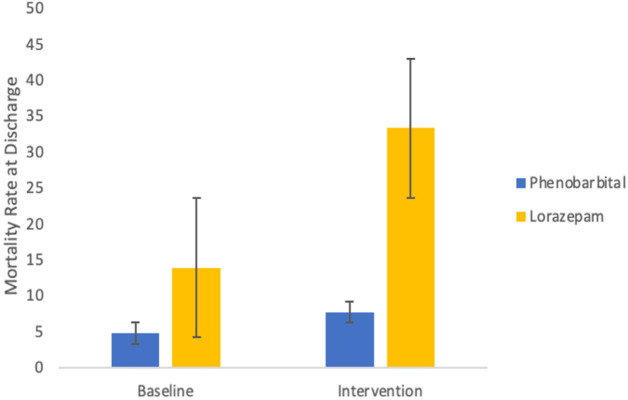
Mortality rate of patients treated with lorazepam versus phenobarbital +/− 1SD during the baseline and intervention periods. For both time periods there was a significantly lower mortality rate in those who received phenobarbital.

The mortality rate among those who received phenobarbital was significantly lower, 4.76%, versus 13.91% compared to those who received only lorazepam (P=0.0008), Figure 3. When patients with cirrhosis were excluded, mortality rate remained significantly higher for those who received lorazepam (p=0.0009).

231 (32.40%) of the ICU patients required mechanical ventilation and had a mean hospital LOS of 18.36 days, mean ICU LOS of 9.79 days. 59 of those received phenobarbital, 152 received only lorazepam and 20 patients received neither. There was no difference in incidence of mechanical ventilation between patients who received phenobarbital (31.22%) versus those who received lorazepam (33.04%, p=0.65).

### Post Intervention

During the 3-month post-intervention period there were 44 patients admitted to the ICU on CIWA protocol, without epilepsy. No patients had a diagnosis of cirrhosis. The mean hospital length of stay (LOS) was 10.92 days (median 7.46 days, SD 9.86), mean ICU LOS 6.14 days (median 3.11 days, SD 9.01).

Of the 44 ICU patients:

– 26 patients (59.01%) received phenobarbital and had a mean hospital LOS of 9.45 days (median 6.92 days, SD 8.58) and a mean ICU LOS of 3.77 days (median 2.59 days, SD 3.59), with an average dose of phenobarbital of 15.60 mg/kg. 15 out of 26 (57.69%) of the patients who received phenobarbital also received lorazepam (average amount 30.90 mg).
– 12 patients (27.27%) received only lorazepam and had a mean hospital LOS of 16.26 days (median 11.92 days, SD 12.67, mean ICU LOS of 9.89 days (median 4.86, SD 13.00), with an average cumulative dose of lorazepam of 11.17 mg. There was no significant difference in ICU LOS for the patients who received phenobarbital versus those who received only lorazepam (p= 0.13, 95% CI: −0.29, 4.28). Hospital LOS was also not significantly different between the groups (p=0.12, 95% CI: −1.21, 15.29). Fifteen (34.01%) of the ICU patients required mechanical ventilation and had a mean hospital LOS of 11.93 days, mean ICU LOS of 5.08 days. 8 of those received phenobarbital, 4 received only lorazepam and 3 received neither. There was no significant difference in mechanical ventilation rates between patients who received phenobarbital versus those who received lorazepam (p=0.94).

The mortality rate among those who received phenobarbital was significantly lower, 7.69%, versus 33.33% among those who received only lorazepam (p=0.04).

Six patients received neither phenobarbital nor lorazepam. Reasons for not receiving those medications included: receiving dexmedetomidine (1), not scoring high enough on CIWA to trigger medication administration (4), and treatment with both propofol and dexmedetomidine (1).

## Discussion

This retrospective observational quality improvement study demonstrated variable practice in the use of benzodiazepines and phenobarbital for the treatment of if AWS in the ICU. Using an educational intervention, we were able to increase the frequency of phenobarbital use for the treatment of AWS.

We reviewed 4 years of trends in lorazepam and phenobarbital use for AWS in one hospital. Over 4 years, as new order sets were incorporated into the electronic medical record, the average amount of phenobarbital increased, and the average amount of lorazepam decreased, average total hospital LOS decreased, while ICU LOS remained steady.

The baseline data revealed significant heterogeneity in medication chosen for AWS treatment, evidenced by the fact that all but 2 of the patients who received phenobarbital also received lorazepam during their hospitalization. Dual treatment with lorazepam and phenobarbital is associated with increased risk of respiratory depression, a side effect of both medications. However, despite the increased risk of respiratory depression, we found no significant difference in intubation rates between patients treated with phenobarbital and lorazepam versus those treated with lorazepam alone during the baseline period. ICU LOS was about 2 days longer, a significant difference, for patients treated with phenobarbital versus those who only received lorazepam. This discrepancy in LOS was likely the result of hospital protocols at the time, requiring patients to stay in the ICU for 24 hours after their last dose of phenobarbital. An unexpected finding during the baseline period was that mortality was significantly higher among patients who were treated with only lorazepam compared to those treated with phenobarbital despite those with phenobarbital having significantly higher mean CIWA scores. Though it is unclear why this occurred, several explanations exist. First, due to the more variable metabolism of lorazepam, more respiratory depression may have occurred. However, if that were the case we would have expected to see a difference between intubation rates between the groups. Second, the patients who received lorazepam may have had more comorbidities; for example, they may have had severe liver disease and therefore phenobarbital was contraindicated. However, a very small number (28) of ICU patients in the baseline period carried a diagnosis of cirrhosis (used as a surrogate for liver disease), and when these patients were excluded from the analysis, mortality remained significantly higher in the lorazepam group. Severity of withdrawal may have also contributed to outcome. However, using mean maximum CIWA score as a surrogate for severity of withdrawal, the patients who received phenobarbital had nearly twice the mean maximum CIWA scores (32) compared to those who received lorazepam (18) suggesting more severe withdrawal in those who were treated with phenobarbital.

Our findings during the baseline period presented an opportunity to develop an initiative to standardize care by delivering training to providers regarding the consistent use of a protocol for the safe and effective use of phenobarbital in the ICU with the goal of maintaining patient safety while decreasing length of stay.

During the 3-month post-intervention period there was still heterogeneity in use of phenobarbital and lorazepam, but patients were more frequently treated with only one of the medications: 1.10% of patients in the baseline period received only phenobarbital; while 37.5% of patients in the post-intervention period were treated with only phenobarbital. Using just phenobarbital or lorazepam has been historically accepted as safer due to theoretical decreased risk of respiratory depression. The increasing percentage of patients who were treated with phenobarbital alone suggests our training initiative was successful in changing prescribing behavior.

During the post-intervention period ICU LOS was not significantly different for those who received phenobarbital compared to those who received lorazepam, which was a change from the baseline period. This finding is likely due to phenobarbital being a safe and effective treatment for alcohol withdrawal combined with changing hospital policies which allowed patients to be treated with phenobarbital and transfer to the floor when clinically stable rather than requiring that patients remain in the ICU for an observation period after the last dose of phenobarbital. A future study including a longer post-intervention observation period is warranted to see if any significant differences in LOS develop.

During the post-intervention period, we again found that the mortality rate among patients who were treated with phenobarbital was significantly lower than those who were treated with lorazepam. This was an unexpected finding and warrants further investigation into possible comorbidities or confounding variables (e.g. liver disease) that may have contributed. The results of this project argue for a randomized controlled trial to ensure that confounding variables are not the cause of the mortality difference we observed.

## Conclusion

Altogether, our findings suggest that a simple intervention aimed at educating physicians regarding the use of an order set in an electronic medical record resulted in behavior change measured by increased use of a phenobarbital order set. Our findings also suggest that phenobarbital may be a safer option for treatment of AWS (lower mortality rate). Though we found no significant post-intervention difference between ICU or hospital LOS related to whether phenobarbital or lorazepam is used, a larger, randomized study is warranted to determine if there is any causal association between the choice of medication used and LOS. Our study is limited by the inability to control for selection bias in the choice of using phenobarbital or lorazepam. However, there are few contraindications to the use of phenobarbital aside from severe liver disease and our results remained unchanged after excluding patients with cirrhosis. We suspect that the choice of pharmacologic treatment may have more to do with prescriber preference than patient characteristics. Future studies should include randomized trials to determine if phenobarbital is a safer and more effective medication than lorazepam for treating AWS.

## References

[1] de Wit M, Jones DG, Sessler CN, Zilberberg MD, Weaver MF (2010). Alcohol-use disorders in the critically ill patient. Chest.

[2] Ghionni N, Abdul Hameed AM, Iriarte BE, Malik A, Maheshwari A, Valentino DJ (2018). Critically ill Caucasian patients admitted for alcohol withdrawal have a longer hospital length of stay and receive higher doses of benzodiazepines. Am J Respir Crit Care Med.

[3] Alvanzo A, Kleinschmidt K, Kmiec JA (2020). The ASAM clinical practice guideline on alcohol withdrawal management. J Addict Med.

[4] Jesse S, Bråthen G, Ferrara M, Keindl M, Ben-Menachem E, Tanasescu R (2017). Alcohol withdrawal syndrome: mechanisms, manifestations, and management. Acta Neurol Scand.

[5] Lindsay DL, Freedman K, Jarvis M, Lincoln P, Williams J, Nelson LS (2020). Executive summary of the American Society of Addiction Medicine (ASAM) clinical practice guideline on alcohol withdrawal management. J Addict Med.

[6] Fuster D, Samet JH (2018). Alcohol use in patients with chronic liver disease. New England Journal of Medicine.

[7] Hendey GW, Dery RA, Barnes RL, Snowden B, Mentler P (2011). A prospective, randomized trial of phenobarbital versus benzodiazepines for acute alcohol withdrawal. Am J Emerg Med.

[8] Nguyen TA, Lam SW (2020). Phenobarbital and symptom-triggered lorazepam versus lorazepam alone for severe alcohol withdrawal in the intensive care unit. Alcohol.

[9] Prabhu S, Geetha HS, Arun Kumar S, Singh G, Gogtay M (2023). Phenobarbital versus Lorazepam for the Management of Alcohol Withdrawal Syndrome (AWS) in Hospitalized Patients: A Retrospective Cohort Study. J Community Med Public Health.

[10] Tidwell WP, Thomas TL, Pouliot JD, Canonico AE, Webber AJ (2018). Treatment of alcohol withdrawal syndrome: phenobarbital vs CIWA-Ar protocol. Am J Crit Care.

